# Association of plasma sphingosine-1-phosphate levels with disease severity and prognosis after intracerebral hemorrhage

**DOI:** 10.3389/fneur.2024.1365902

**Published:** 2024-04-03

**Authors:** Xuan Yang, Kaixin Wang, Ping Shen, Tong Zhou, Yudi Xu, Yufei Chen, Yanfei Li, Yaobing Yao, Zhe Gong, Ranran Duan, Lijun Jing, Yanjie Jia

**Affiliations:** ^1^Department of Neurology, The First Affiliated Hospital of Zhengzhou University, Zhengzhou, China; ^2^Department of Neurology, Xinzheng Huaxin Minsheng Hospital, Zhengzhou, Henan, China; ^3^Department of Neurology, Huaiyang County People’s Hospital, Zhoukou, Henan, China

**Keywords:** sphingosine-1-phosphate, spontaneous intracerebral hemorrhage, severity, prognosis, inflammation

## Abstract

**Purpose:**

Sphingosine-1-phosphate (S1P) is a signaling lipid involved in many biological processes, including inflammatory and immune regulatory responses. The study aimed to determine whether admission S1P levels are associated with disease severity and prognosis after spontaneous intracerebral hemorrhage (ICH).

**Methods:**

Data of 134 patients with spontaneous ICH and 120 healthy controls were obtained from Biological Resource Sample Database of Intracerebral Hemorrhage at the First Affiliated Hospital of Zhengzhou University. Plasma S1P levels were measured. Regression analyses were used to analyze the association between S1P levels and admission and 90-day modified Rankin scale (mRS) scores. Receiver operating characteristic (ROC) curves assessed the predictive value of S1P levels for ICH severity and prognosis.

**Results:**

Patients with ICH exhibited elevated plasma S1P levels compared to the control group (median 286.95 vs. 239.80 ng/mL, *p* < 0.001). When divided patients into mild-to-moderate and severe groups according to their mRS scores both at admission and discharge, S1P levels were significantly elevated in the severe group compared to the mild-to-moderate group (admission 259.30 vs. 300.54, *p* < 0.001; 90-day 275.24 vs. 303.25, p < 0.001). The patients were divided into three groups with different concentration gradients, which showed significant statistical differences in admission mRS scores (3 vs. 4 vs. 5, *p* < 0.001), 90-day mRS scores (2.5 vs. 3 vs. 4, *p* < 0.001), consciousness disorders (45.5% vs. 68.2% vs. 69.6%, *p* = 0.033), ICU admission (29.5% vs. 59.1% vs. 89.1%, *p* < 0.001), surgery (15.9% vs. 47.7% vs. 82.6%, *p* < 0.001), intraventricular hemorrhages (27.3% vs. 61.4% vs. 65.2%, *p* < 0.001) and pulmonary infection (25% vs. 47.7% vs. 84.8%, *p* < 0.001). Multivariate analysis displayed that S1P level was an independent risk factor for disease severity (OR = 1.037, 95% CI = 1.020–1.054, *p* < 0.001) and prognosis (OR = 1.018, 95% CI = 1.006–1.030, *p* = 0.003). ROC curves revealed a predictive value of S1P levels with an area under the curve of 0.7952 (95% CI = 0.7144–0.8759, *p* < 0.001) for disease severity and 0.7105 (95% CI = 0.6227–0.7983, *p* < 0.001) for prognosis.

**Conclusion:**

Higher admission S1P is associated with worse initial disease severity and 90-day functional outcomes in intracerebral hemorrhage.

## Introduction

Acute, spontaneous intracerebral hemorrhage (ICH) is the second most common form of stroke, affecting approximately two million people worldwide every year (1, 2), which is characterized by high incidence rate, high disability rate, high mortality, while few therapeutic interventions available (3). The inflammatory response after ICH is caused by the components of the hematoma, which in turn can exacerbate the damage caused by ICH (4). The occupying effect and systemic inflammatory response after the formation of intracranial hematoma can lead to secondary neurological deterioration, so inflammatory response is an important part of secondary brain injury (5). The severity of inflammatory reactions can be evaluated by clinical manifestations and biochemical inflammatory markers such as neutrophils, lymphocytes, CRP, NLR, TNF-α, IL-6 and MMP-9 (6–10). Increasing evidence declares that systemic inflammation secondary to stroke can increase the risk of exacerbating stroke and worsen the clinical prognosis of stroke patients (11). Analyzing these peripheral markers of central inflammation can provide reference for potential pathophysiology and may provide insights for clinical practice and future research.

Sphingosine-1-phosphate (S1P) is a signaling lipid involved in many biological processes, including inflammatory and immune regulatory responses. It is produced through the phosphorylation of sphingosine by the sphingosine kinases SPHK1 and SPHK2. The circulating S1P in the blood mainly comes from activated platelets, blood cells, and endothelial cells (12–14), and exists at high concentrations in the plasma, mainly binding to albumin or high-density lipoprotein (HDL) (15). S1P serves as a ligand for a family of specific high-affinity G protein-coupled lipid cell surface receptors (S1PR) and combines with them to exert various physiological effects. Evidence suggests that S1P mediates assorted and complicated roles in atherosclerosis, myocardial infarction, and stroke by regulating endothelial function, inflammatory responses, and immune cell behavior (16). Therefore, S1P and different S1P related treatments have been studied in numerous experimental stroke models and clinical practice (17–20). However, to the best of our knowledge, there is still a lack of multicenter large-scale prospective clinical studies on the correlation between S1P levels and disease severity and prognosis in acute ICH patients. Therefore, in the present study, we evaluated the S1P levels and analyzed whether S1P levels at admission were interrelated with disease severity and prognosis after ICH.

## Methods

### Patients

The study protocol was approved by the Ethics Review Committee of Zhengzhou University (2023-KY-0242). The basic data survey on cerebral hemorrhage incidence and risk factors in Central China was a special project of the Ministry of Science and Technology of the People’s Republic of China. The trial enrolled patients with spontaneous ICH from January 2018 to December 2022. The samples for this retrospective study were all from the biological sample bank of the First Affiliated Hospital of Zhengzhou University. Each patient, or their legally authorized representative, provided written informed consent for participation in the trial. The inclusion criteria of ICH group were as follows: (1) age > 14 years; (2) admission within 24 h of symptom onset, with the diagnosis of ICH confirmed on imaging; (3) informed consent from the participant or legally authorized representative. Exclusion criteria: (1) asymptomatic patients with physical signs of cerebral hemorrhage; (2) other types of cerebral hemorrhage, such as cerebral infarction with cerebral hemorrhage, subarachnoid hemorrhage, traumatic cerebral hemorrhage and venous cerebral hemorrhage; (3) presence of other diseases that may affect S1P level, such as serious infection, tumor, systemic immune disease; (4) patients with incomplete clinical data; (5) use of drugs that can affect serum S1P levels, such as Fingolimod and Siponimod; (6) Unable to obtain follow-up data. According to the inclusion and exclusion criteria, other non-inflammatory neurological disease patients who were hospitalized at the same time as ICH patients were selected as controls group, including dizziness, headache, somatization disorders, et al. The exclusion criteria were as follows: (1) Age ≤ 14 years old; (2) the clinical data is incomplete; (3) other comorbidities or history of drug use that affect S1P levels.

### Biological index determination

Blood samples from the controls and patients were processed in identical manner. After collection, extraction, and recording, the blood samples were stored in the biological sample bank of the First Affiliated Hospital of Zhengzhou University through cold-chain transport. Following centrifugation at 4000 rpm for 10 min within 1 h collection, all samples are stored in the −80°C refrigerator until they are centrally retrieved from the sample bank for blind detection. The S1P concentration was determined using an ELISA Kit (Fine-Test, Wuhan). The tests were conducted in accordance with the manufacturer’s protocols. Standard curves were drawn using Curve Expert 1.3 for each measurement and the results were reported in ng/ml values.

### Data collection

The data collected in this retrospective study were collected through the hospital’s electronic medical record system and included baseline information, clinical symptoms, laboratory examinations (routine blood tests, hepatorenal function, blood clotting function, erythrocyte sedimentation rate, and C-reactive protein level), hospital stay, ICU admission, surgery, pulmonary infection and imaging findings at admission. All routine clinical assays were conducted at the Biochemistry Laboratory of First Affiliated Hospital of Zhengzhou University. The investigators were blinded to all clinical information. At least two experienced professional neurologists independently assessed modified Rankin Scale (mRS) scores of all participants based on disease course record at admission. Patients were followed up until death or 90 days after ICH by using case data or structured telephone interview and assessment of 90-day neurological functional status was fulfilled utilizing modified Rankin scale (mRS).

### Grouping

Convert continuous variable into categorical variables. Patients were divided into the mild-to-moderate group (0–3) and the severe group (4–6) according to mRS scores, and divided into three groups--low, medium, and high concentration based on S1P concentration to identify predictive factors for the severity and prognosis after ICH.

### Statistical analysis

Data were analyzed using the SPSS software (version 26.0; International Business Machines Corporation, Chicago, IL, USA). Graphs were produced using GraphPad Prism 9.5 (GraphPad Inc., La Jolla, CA, USA). All variables underwent normal distribution testing, and normally distributed data are expressed as the mean ± standard deviation, non-normally distributed data were showed as median with interquartile range (IQR). Differences between two groups were tested using either *t*-tests for two groups or the non-parametric Mann–Whitney U test. The Kruskal-Wallis test was adopted to compare multiple classification data groups. Categorical data are presented as the number of cases (percentage) and were compared using Chi-squared or Fisher’s exact tests. Logistic regression analysis was used to evaluate the factors potentially related to severe neurological deficits and poor outcomes by computing odds ratios (OR) with 95% confidence intervals (CIs). Variables with a significance level of *p* < 0.1 in the univariate logistic regression analysis and those that were closely related to dependent variables in the clinic were included in the multivariate model. A receiver operating characteristic (ROC) curve was used to estimate the predictive value of S1P levels for disease severity and functional outcomes. A correlation analysis was performed using the Spearman correlation analysis. For all tests, a *p*-value of <0.05 was considered significant.

## Results

### Clinical characteristics of the patients with ICH and healthy individuals

This cohort study included 134 patients with ICH and 120 healthy individuals. Among the ICH group 51 (38.1%) were female patients with an average onset age of 57.0 ± 14.3 years. In the control group, 53 female patients (44.2%) aged 54.9 ± 11.2 years on average were included. Between the two groups, there were no significant differences in age or sex (*p* > 0.05), expressing comparability. The incidence of diabetes, hypertension, smoking and drinking did not differ significantly between the two groups (*p* > 0.05). Our results indicated that S1P (median 286.95 vs. 239.80 ng/mL, *p* < 0.001) were higher in ICH patients than in control group. Our study found that apart from platelet count and monocytes, there were statistically significant differences in other blood indicators between the two groups. Blood WBC count (median 10.36 vs. 5.84 × 109/L, *p* < 0.001), neutrophil count (median 8.81 vs. 3.30 × 109/L, *p* < 0.001), NLR (median 11.26 vs. 1.74, *p* < 0.001), PLR (median 241.14 vs. 114.91, *p* < 0.001), PT (median 11.4 vs. 10.3 s, *p* < 0.001), fibrinogen (median 3.27 vs. 2.4 g/L, *p* < 0.001) and D-dimer (median 0.665 vs. 0.06 mg/L, *p* < 0.001) were higher in patients with ICH than in healthy individuals (Table 1).

**Table 1:** Demographic and clinical characteristics of the patients with ICH and control.

	Patients with ICH (*n* = 134)	Control	*p*
		(*n* = 120)	
Demographic data			
Age onset, years, mean ± SD	57.0 ± 14.3	54.9 ± 11.2	0.187
Sex, female, n%	51 (38.1%)	53 (44.2%)	0.323
Smoking, n (%)	39 (29.1%)	18 (15%)	0.007
Drink, n (%)	43 (32.1%)	27 (22.5%)	0.114
Hypertension, n (%)	63 (47.0%)	54 (45.0%)	0.748
Diabetes, n (%)	19 (14.2%)	14 (11.7%)	0.552
Laboratory test results			
S1P, mean ± SD, ng/ml	286.95 ± 39.92	239.80 ± 59.00	<0.001*
RBC, median (IQR), × 1,012/L	4.16 (3.73–4.58)	4.45 (4.17–4.84)	<0.001*
WBC, median (IQR), × 109/L	10.36 (7.45–13.33)	5.84 (5.07–6.87)	<0.001*
Hemoglobin, median (IQR), g/L	128.35 (114.10–142.33)	136.50 (125.00–147.00)	<0.001*
Platelet, median (IQR), × 109/L	202 (147.75–259.25)	223 (186.75–270.75)	0.016*
Neutrophil, median (IQR), × 109/L	8.81 (5.42–11.93)	3.30 (2.55–4.00)	<0.001*
Monocytes, median (IQR), × 109/L	0.50 (0.34–0.70)	0.41 (0.35–0.52)	0.008*
Lymphocytes, median (IQR), × 109/L	0.82 (0.58–1.15)	1.94 (1.65–2.31)	<0.001*
Eosinophil, median (IQR), × 109/L	0.02 (0–0.09)	0.11 (0.07–0.17)	<0.001*
Basophil, median (IQR), × 109/L	0.02 (0.01–0.03)	0.03 (0.02–0.04)	<0.001*
NLR, median (IQR)	11.26 (5.14–18.96)	1.74 (1.24–2.16)	<0.001*
LMR, median (IQR)	1.64 (1.01–2.65)	4.59 (3.55–5.90)	<0.001*
PLR, median (IQR)	241.14 (175.72–339.95)	114.91 (94.83–142.56)	<0.001*
PT, median (IQR), s	11.4 (10.7–12.1)	10.3 (9.9–10.9)	<0.001*
APTT, median (IQR), s	28.2 (26.1–31.2)	30.8 (28.6–34.2)	<0.001*
FIB, median (IQR), g/L	3.27 (2.69–4.20)	2.40 (2.16–2.89)	<0.001*
TT, median (IQR), s	13.95 (13.07–15.32)	15.05 (14.15–16.10)	<0.001*
D-Dimer, median (IQR), mg/L	0.665 (0.205–1.878)	0.060 (0.037–0.090)	<0.001*

S1P, sphingosine-1-phosphate; WBC, white blood cell; RBC, red blood cell; LMR, lymphocyte–monocyte ratio; NLR, neutrophil–lymphocyte ratio; PLR, platelet–lymphocyte ratio; PT, prothrombin time; APTT, activated partial thrombin time; TT, thrombin time; FIB, fibrinogen.

*p < 0.05.

### Clinical characteristics of the ICH patients with different severity and prognosis

In Table 2, patients were divided into mild to moderate group (*n* = 41) and severe group (*n* = 93) based on the mRS score at admission. Summarizing and comparing the laboratory test results of the two groups, S1P (median 300.54 vs. 259.30 ng/mL, *p* < 0.001), lymphocytes count (median 0.9 vs. 0.7 × 109/L, *p* = 0.019), and CRP (median 16.23 vs. 8.11 mg/L, *p* = 0.002) in the severe group was higher than that in the mild to moderate group. We observed no remarkable differences between the groups concerning sex, age, smoking, drinking, or prevalence of diabetes, hypertension, RBC, WBC, hemoglobin, platelet, neutrophil, monocytes, eosinophil, basophil, NLR, LMR, PLR, coagulation function and ESR.

**Table 2:** Clinical characteristics of the ICH patients with different severity.

	Total	Mild to moderate group	Severe group	*p*
	(*n* = 134)	(*n* = 41)	(*n* = 93)	
Demographic data				
Age onset, years, mean ± SD	57.0 ± 14.3	53.9 ± 13.0	58.4 ± 14.6	0.089
Gender, female, n%	51 (38.1%)	18 (43.9%)	33 (35.5%)	0.355
Smoking, n (%)	39 (29.1%)	13 (31.7%)	26 (28.0%)	0.660
Drink, n (%)	43 (32.1%)	13 (31.7%)	30 (32.3%)	0.950
Hypertension, n (%)	63 (47.0%)	26 (63.4%)	57 (61.3%)	0.815
Diabetes, n (%)	19 (14.2%)	8 (19.5%)	11 (11.8%)	0.240
Laboratory test results				
S1P, mean ± SD, ng/ml	286.95 ± 39.92	259.30 ± 35.14	300.54 ± 35.16	<0.001*
RBC, median (IQR), ×1,012/L	4.16 (3.74 ~ 4.56)	4.23 (3.64 ~ 4.62)	4.14 (3.74 ~ 4.57)	0.548
WBC, median (IQR), ×109/L	10.36 (7.46 ~ 13.33)	9.37 (6.13 ~ 12.04)	10.70 (7.72 ~ 14.55)	0.076
Hemoglobin, median (IQR), g/L	126.4 ± 22.8	128 (113.15 ~ 140.25)	130 (113.80 ~ 143.20)	0.860
Platelet, mean ± SD, × 109/L	211.4 ± 90.4	197.8 ± 91.3	217.4 ± 89.8	0.249
Neutrophil, mean ± SD, × 109/L	9.07 ± 4.37	8.42 ± 3.75	9.00 ± 4.58	0.099
Monocytes, median (IQR), × 109/L	0.51 (0.34 ~ 0.70)	0.43 (0.31 ~ 0.66)	0.51 (0.37 ~ 0.71)	0.288
Lymphocytes, median (IQR), × 109/L	0.83 (0.58 ~ 1.16)	0.70 (0.56 ~ 0.97)	0.90 (0.6 ~ 1.3)	0.019*
Eosinophil, median (IQR), × 109/L	0.02 (0 ~ 0.09)	0.03 (0 ~ 0.11)	0.02 (0 ~ 0.06)	0.323
Basophil, median (IQR), × 109/L	0.02 (0.01 ~ 0.03)	0.01 (0 ~ 0.03)	0.02 (0.01 ~ 0.03)	0.316
NLR, median (IQR)	11.26 (5.14 ~ 18.96)	11.59 (5.66 ~ 16.28)	11.06 (5.05 ~ 19.74)	0.757
LMR, median (IQR)	1.64 (1.01 ~ 2.65)	1.63 (0.98 ~ 2.43)	1.71 (1.07 ~ 2.72)	0.548
PLR, median (IQR)	241.14 (175.72 ~ 339.95)	268.52 (189.53 ~ 344.96)	229.20 (162.54 ~ 336.92)	0.197
PT, median (IQR), s	11.4 (10.7 ~ 12.1)	11.6 (10.9 ~ 12.1)	11.3 (10.7 ~ 12.2)	0.379
APTT, median (IQR), s	28.25 (26.10 ~ 31.20)	28.30 (26.25 ~ 31.40)	28.20 (25.90 ~ 30.85)	0.460
FIB, median (IQR), g/L	3.28 (2.70 ~ 4.20)	3.35 (2.67 ~ 4.23)	3.09 (2.70 ~ 4.21)	0.969
TT, median (IQR), s	13.95 (13.08 ~ 15.33)	14.20 (13.40 ~ 16.50)	13.70 (12.90 ~ 15.25)	0.196
D-Dimer, median (IQR), mg/L	0.665 (0.205 ~ 1.878)	0.770 (0.155 ~ 2.335)	0.590 (0.225 ~ 1.705)	0.843
ESR, median (IQR), mm/h	14.0 (6.0 ~ 42.0)	10.0 (5.1 ~ 28.0)	18.0 (7.0 ~ 44.4)	0.093
CRP, median (IQR), mg/L	13.9 (4.6 ~ 28.6)	8.11 (2.69 ~ 17.65)	16.23 (6.86 ~ 34.70)	0.002*

mRS, modified Rankin scale; IQR, interquartile range; RBC, red blood cell; APTT, activated partial thromboplastin time; FIB, fibrinogen; TT, thrombin time; CRP, C-reactive protein; ESR, erythrocyte sedimentation rate.

*p < 0.05.

To determine the factors affecting disease prognosis, we compared the mild to moderate and severe groups based on 90-day mRS. As shown in Table 3, except for that S1P (median 303.25 vs. 275.24 ng/mL, *p* < 0.001) and CRP (median 19.73 vs. 10.62 mg/L, *p* = 0.005) in the severe group was higher than that in the mild to moderate group, there was no statistically significant difference in demographic and other laboratory indicators between the two groups.

**Table 3:** Clinical characteristics of the ICH patients with different prognosis.

	Mild to moderate group	Severe group	*p*
	(*n* = 41)	(*n* = 93)	
Demographic data			
Age onset, years, mean ± SD	55.5 ± 12.1	59.2 ± 16.7	0.145
Gender, female, n%	31 (39.7%)	20 (35.7%)	0.636
Smoking, n (%)	26 (33.3%)	13 (23.2%)	0.203
Drink, n (%)	26 (33.3%)	17 (30.4%)	0.716
Hypertension, n (%)	46 (59.0%)	37 (66.1%)	0.404
Diabetes, n (%)	12 (15.4%)	7 (12.5%)	0.637
Laboratory test results			
S1P, mean ± SD, ng/ml	275.24 ± 39.01	303.25 ± 35.47	<0.001*
RBC, median (IQR), ×1,012/L	4.16 (3.87 ~ 4.64)	4.15 (3.54 ~ 4.55)	0.371
WBC, median (IQR), ×109/L	10.1 (6.86 ~ 13.26)	10.38 (8.32 ~ 13.48)	0.339
Hemoglobin, median (IQR), g/L	131.50 (115.63 ~ 142.33)	126.00 (111.30 ~ 142.78)	0.367
Platelet, mean ± SD, × 109/L	210.6 ± 91.6	212.5 ± 89.5	0.908
Neutrophil, mean ± SD, × 109/L	8.79 ± 4.29	9.45 ± 4.50	0.393
Monocytes, median (IQR), × 109/L	0.48 (0.33 ~ 0.62)	0.53 (0.40 ~ 0.77)	0.062
Lymphocytes, median (IQR), × 109/L	0.79 (0.56 ~ 1.13)	0.87 (0.60 ~ 1.28)	0.312
Eosinophil, median (IQR), × 109/L	0.02 (0 ~ 0.09)	0.02 (0 ~ 0.06)	0.838
Basophil, median (IQR), × 109/L	0.02 (0.01 ~ 0.03)	0.01 (0.01 ~ 0.03)	0.345
NLR, median (IQR)	11.23 (5.11 ~ 17.21)	11.35 (5.44 ~ 19.77)	0.951
LMR, median (IQR)	1.74 (1.08 ~ 2.66)	1.48 (0.98 ~ 2.65)	0.386
PLR, median (IQR)	249.61 (176.78 ~ 351.49)	226.40 (163.58 ~ 336.36)	0.426
PT, median (IQR), s	11.4 (10.7 ~ 12.0)	11.4 (10.7 ~ 12.4)	0.876
APTT, median (IQR), s	28.35 (26.18 ~ 31.20)	27.95 (25.95 ~ 31.20)	0.773
FIB, median (IQR), g/L	3.34 (2.73 ~ 4.25)	3.13 (2.67 ~ 3.88)	0.545
TT, median (IQR), s	13.75 (13.18 ~ 15.75)	14.10 (12.83 ~ 15.15)	0.585
D-Dimer, median (IQR), mg/L	0.665 (0.210 ~ 1.792)	0.620 (0.160 ~ 2.548)	0.584
ESR, median (IQR), mm/h	17.0 (6.0 ~ 42.0)	13.0 (6.25 ~ 44.0)	0.882
CRP, median (IQR), mg/L	10.62 (3.61 ~ 20.76)	19.73 (9.82 ~ 35.34)	0.005*

mRS, modified Rankin scale; IQR, interquartile range; RBC, red blood cell; APTT, activated partial thromboplastin time; FIB, fibrinogen; TT, thrombin time; CRP, C-reactive protein; ESR, erythrocyte sedimentation rate.

*p < 0.05.

According to the concentration values of plasma S1P, patients were divided into three groups with different concentration gradients: low concentration group (median 250.04, *n* = 44), medium concentration group (median 286.44, *n* = 44), and high concentration group (median 320.97, *n* = 46). We found that there were statistically significant differences in consciousness disorders (45.5% vs. 68.2% vs. 69.6%, *p* = 0.033), ICU admission (29.5% vs. 59.1% vs. 89.1%, *p* < 0.001), surgery (15.9% vs. 47.7% vs. 82.6%, *p* < 0.001), pulmonary infection (25% vs. 47.7% vs. 84.8%, *p* < 0.001), mRS scores at admission (median 3 vs. 4 vs. 5, *p* < 0.001) and 90-days mRS score (median 2.5 vs. 3 vs. 4, *p* < 0.001) among different concentration groups. The three groups also showed a certain trend in mortality rate (0 vs. 2.2% vs. 6.5%). When analyzing the imaging indicators, no significant differences in the location of cerebral hemorrhage were detected among the three groups. However, patients with higher concentration of S1P had a higher proportion of intraventricular hemorrhages (IVH) (27.3% vs. 61.4% vs. 65.2%, *p* < 0.001) (Table 4).

**Table 4:** Demographic and clinical data of cerebral hemorrhage patients with different concentrations of S1P.

	Total	Low concentration group	Middle concentration group	High concentration group	*p*
	(*n* = 134)	(*n* = 44)	(*n* = 44)	(*n* = 46)	
S1P, median (IQR), ng/ml	286.90 (262.18 ~ 313.00)	250.04 (226.00 ~ 262.18)	286.44 (276.85 ~ 292.22)	320.97 (311.73 ~ 349.19)	–
Age onset, mean ± SD, years	57.0 ± 14.3	53.91 ± 12.78	56.98 ± 14.99	60.09 ± 14.60	0.112
Sex, female, n%	51 (38.1%)	15 (34.1%)	19 (43.2%)	17 (37.0%)	0.668
Smoking, n (%)	39 (29.1%)	13 (29.5%)	13 (28.3%)	13 (29.5%)	0.988
Drink, n (%)	43 (32.1%)	11 (25.0%)	13 (29.5%)	19 (41.3%)	0.230
Hypertension, n (%)	83 (61.9%)	25 (56.8%)	31 (70.5%)	27 (58.7%)	0.359
Diabetes, n (%)	19 (14.2%)	5 (11.4%)	9 (20.4%)	5 (10.9%)	0.346
Headache, n (%)	41 (30.6%)	13 (29.5%)	13 (29.5%)	15 (32.6%)	0.935
Vomiting, n (%)	73 (54.5%)	19 (43.2%)	23 (52.3%)	31 (67.4%)	0.066
Consciousness disorders, n (%)	82 (61.2%)	20 (45.5%)	30 (68.2%)	32 (69.6%)	0.033*
Seizures, n (%)	8 (6.0%)	2 (4.5%)	3 (6.5%)	3 (6.8%)	0.882
Limb weakness, n (%)	76 (56.7%)	22 (50.0%)	28 (63.6%)	26 (56.5%)	0.434
ICH Location, n (%)					0.876
Lobar	11 (8.2%)	4 (9.1%)	4 (9.1%)	3 (6.5%)	
Non-lobar	123 (88.1%)	40 (90.9%)	40 (90.9%)	43 (93.5%)	
IVH, n (%)	74 (55.2%)	12 (27.3%)	27 (61.4%)	30 (65.2%)	<0.001*
Other clinical characteristics					
Admission mRS score, median (IQR)	4 (3–5)	3 (3–4)	4 (3–5)	5 (4–5)	<0.001*
90-days mRS score, median (IQR)	3 (3–4)	2.5 (2–3)	3 (3–4)	4 (3–5)	<0.001*
Hospital stays, median (IQR), days	10.5 (4.0–21.3)	7.5 (2–21)	14.5 (4–29.0)	10.0 (4–21.0)	0.399
Surgery, n (%)	66 (58.2%)	7 (15.9%)	21 (47.7%)	38 (82.6%)	<0.001*
ICU admission, n (%)	80 (59.7%)	13 (29.5%)	26 (59.1%)	41 (89.1%)	<0.001*
Pulmonary infection, n (%)	71 (53%)	11 (25%)	21 (47.7%)	39 (84.8%)	<0.001*
Death, n (%)	4 (2.9%)	0	1 (2.2%)	3 (6.5%)	-

ICU, intense care unit; IVH, intraventricular hemorrhage.

*p < 0.05.

### Predictors associated with severity and prognosis of patients with ICH

**Figure 1:**
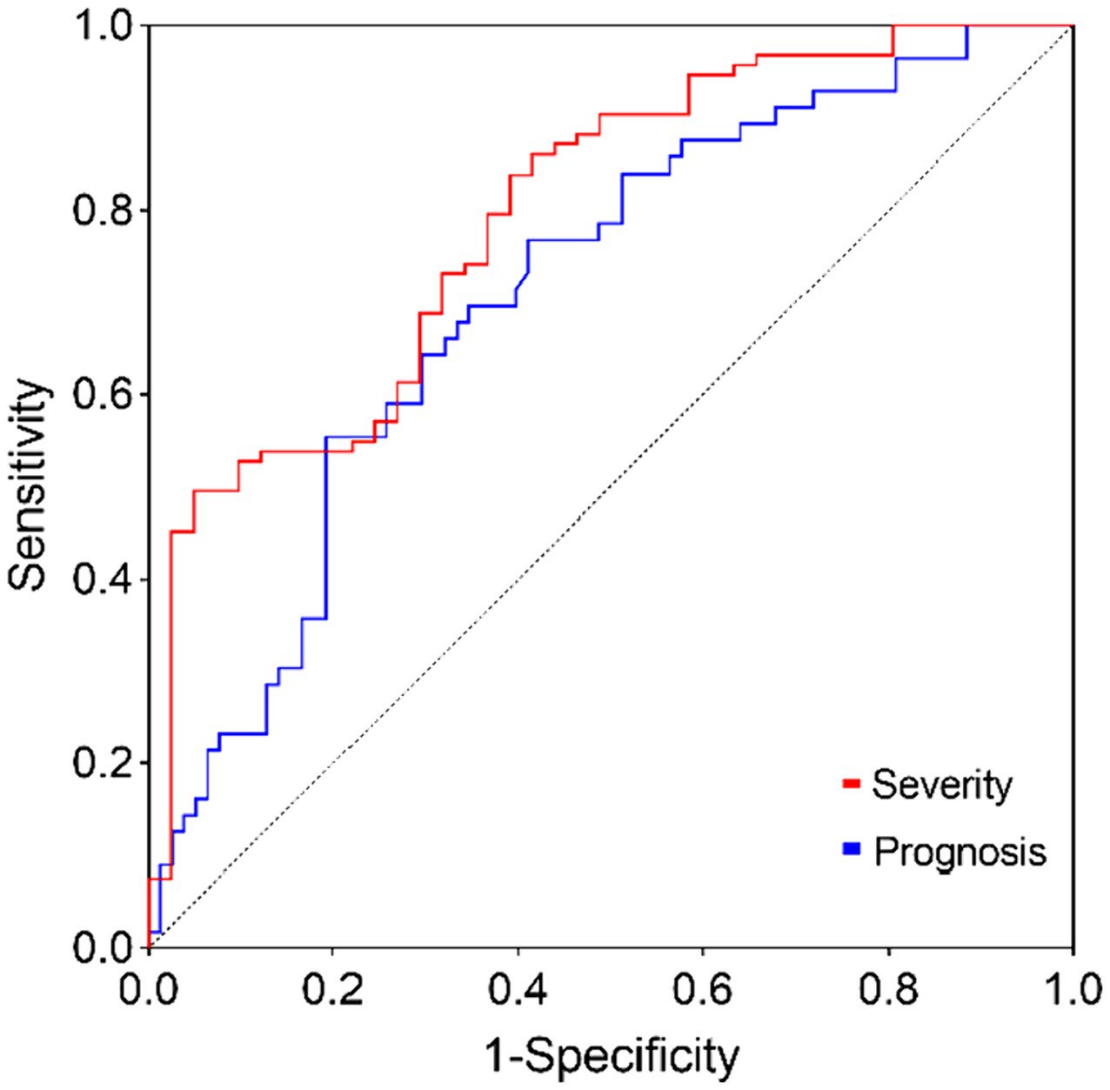
The ROC curve of S1P predicts the severity and prognosis of ICH.

Tables 5, 6 analyze the potential influencing factors on disease severity and prognosis. Univariate logistic regression analysis revealed significant correlations between disease severity and variables like S1P levels (OR = 1.036, 95% CI = 1.021–1.050, *p* < 0.001), CRP (OR = 1.026, 95% CI = 1.004–1.049, *p* = 0.023), lymphocytes (OR = 3.269, 95% CI = 1.238–8.628, *p* = 0.017), and IVH (OR = 2.400, 95% CI = 1.125–5.118, *p* = 0.023). In the multivariate model, S1P level (OR = 1.037, 95% CI = 1.020–1.054, *p* < 0.001) emerged as an independent risk factor for disease severity. Furthermore, logistic regression analysis of disease prognosis showed that S1P level (OR = 1.018, 95% CI = 1.006–1.030, *p* = 0.003) was an independent risk factor for the prognosis of cerebral hemorrhage. Predictive value of S1P levels was assessed using ROC curves for disease severity and prognosis. The area under the curve was 0.7952 (95% CI = 0.7144–0.8759, *p* < 0.001) for disease severity and 0.7105 (95% CI = 0.6227–0.7983, *p* < 0.001) for prognosis (Figure 1).

**Table 5:** Binary logistic regression of disease severity in patients with ICH.

	Univariate analysis		Multivariable analysis	
	OR (95% CI)	*p*	OR (95% CI)	*p*
Age onset	1.023 (0.996–1.051)	0.092	1.011 (0.980–1.044)	0.482
Sex	0.703 (0.332–1.486)	0.356		
S1P	1.036 (1.021–1.050)	<0.001	1.037 (1.020–1.054)	<0.001*
CRP	1.026 (1.004–1.049)	0.023	1.006 (0.985–1.028)	0.556
Lymphocytes	3.269 (1.238–8.628)	0.017	0.560 (0.194–2.179)	0.485
IVH	2.400 (1.125–5.118)	0.023	0.989 (0.399–2.452)	0.981
ICH Location	2.089 (0.431–10.129)	0.360		

CRP, C-reactive protein; ESR, erythrocyte sedimentation rate; IVH, intraventricular hemorrhage.

*p < 0.05.

**Table 6:** Binary logistic regression of disease prognosis in patients with ICH.

	Univariate analysis		Multivariable analysis	
	OR (95% CI)	*p*	OR (95% CI)	*p*
Age onset	1.019 (0.994–1.044)	0.147		
Sex	0.842 (0.414–1.714)	0.636		
S1P	1.020 (1.010–1.031)	<0.001	1.018 (1.006–1.030)	0.003*
CRP	1.013 (0.998–1.029)	0.084	1.000 (0.983–1.018)	0.957
Lymphocytes	1.608 (0.752–3.440)	0.221		
IVH	2.799 (1.371–5.716)	0.005	1.850 (0.856–3.998)	0.118
ICH Location	0.284 (0.059–1.369)	0.117		

CRP, C-reactive protein; ESR, erythrocyte sedimentation rate; IVH, intraventricular hemorrhage.

*p < 0.05.

### Correlation between S1P levels and other laboratory indexes in ICH

**Figure 2:**
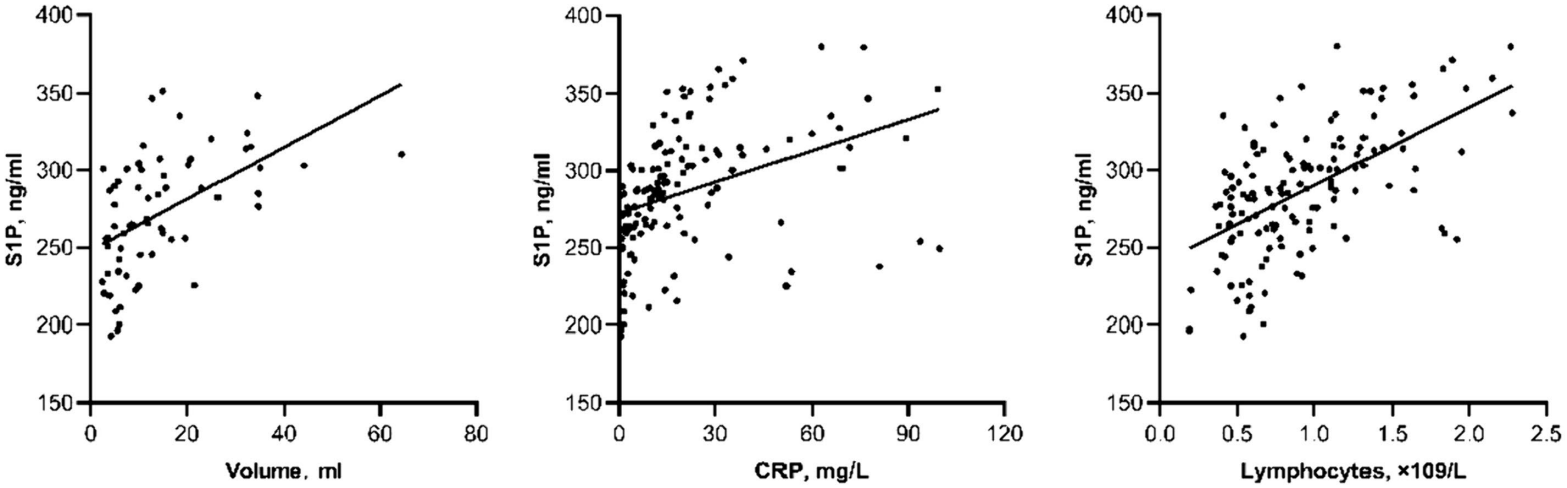
Scatter plot of correlation between other laboratory indicators and S1P level in actue ICH.

To analyze the factors influencing changes in plasma S1P concentration after acute cerebral hemorrhage, a Spearman correlation analysis was performed. The results showed a significant correlation between S1P levels in acute cerebral hemorrhage and bleeding volume (*r* = 0.583, *p* < 0.001), CRP (*r* = 0.567, *p* < 0.001), and lymphocyte count (*r* = 0.548, *p* < 0.001) (Figure 2).

### ICH location modifies the association between S1P and ICH

According to the stratified analysis of the location of ICH, we found a stronger correlation between the admission level of S1P in patients with non-lobar ICH and disease severity (unadjusted: OR = 1.041, 95% CI = 1.024–1.058, *p* < 0.001; adjusted model: OR = 1.043, 95% CI = 1.025–1.060, *p* < 0.001). However, in the analysis of disease prognosis, the position of ICH did not show a stronger correlation (unadjusted: OR = 1.019, 95% CI = 1.008–1.029, *p* = 0.001; adjusted model: OR = 1.018, 95% CI = 1.007–1.029, *p* = 0.001). We also found a higher correlation between S1P levels and ICH volume in patients with deep hemorrhages (*r* = 0.646, *p* < 0.001).

## Discussion

In this retrospective study, we investigated the possible association between early S1P levels and disease severity and prognosis by compiling a large dataset of patients with spontaneous cerebral hemorrhage. We found that higher levels of S1P were associated with higher disease severity at admission and poorer prognosis. Meanwhile, high concentrations of S1P were also associated with high surgical outcomes, pulmonary infections, intraventricular hemorrhage, and ICU occupancy rates. Further logistical analysis suggests that S1P may be an independent risk factor for the severity and prognosis of cerebral hemorrhage, and its risk increases with increasing concentration. In addition, the S1P level in patients with cerebral hemorrhage is positively correlated with the amount of cerebral hemorrhage, lymphocyte count, and CRP level.

Preclinical and clinical studies have emphasized the role of inflammation in neuronal death and neurological dysfunction after ICH (21). Recent studies have further shown that brain inflammation induced by ICH can lead to opposite local and systemic effects. In our study, we also observed significant differences in routine blood tests, coagulation function, and inflammatory markers between the ICH group and the healthy control group, which is consistent with the results of other studies (22–24). After ICH, the blood–brain barrier (BBB) is significantly disrupted, accompanied by neuroinflammatory reactions and the formation of vascular edema. Many cytokines, such as IL-23, IL-4, IL-10, and TGF-β, can be released from neurons, glial cells, or infiltrating macrophages, lymphocytes, and neutrophils in the ischemic or hemorrhagic brain into peripheral blood, further regulating the activation and polarization of white blood cells and other inflammatory cells (25–28). For example, the neutrophil-to-lymphocyte ratio (NLR) is supposed to an important parameter for estimating systemic inflammatory status and infection risk (29). The platelet-to-lymphocyte ratio (PLR) is an indicator of platelet aggregation and systemic inflammation (30). We found that the NLR and PLR in the cerebral hemorrhage group were significantly higher than those in the healthy group, and the lymphocyte-to-monocyte ratio (LMR) was lower than that in the control group, suggesting that patients with cerebral hemorrhage have a systemic inflammatory response in addition to the inflammatory response around the local hematoma. Conversely, systemic cellular and molecular immune responses probably also affect the local immune response to the hematoma and the prognosis of patients with ICH (31).

The focus of this study is to explore the relationship between S1P levels and the severity and prognosis of cerebral hemorrhage. We found that compared to healthy individuals, the levels of S1P in the plasma of patients with cerebral hemorrhage increased in a short period of time, and S1P was associated with the severity of admission and prognosis. A multiple regression analysis with the severity of the patient at admission as the dependent variable showed that for every 1 ng/mL increase in serum S1P concentration, the risk of severe illness increased by 1.030%. Prognostic analysis shows that for every increase in serum S1P concentration of 1 ng/mL, the risk of developing serious diseases increases by 1.015%. To our knowledge, there is no direct evidence to suggest a correlation between circulating S1P levels, disease severity, and prognosis. Therefore, we can only speculate on the possible causes. After cerebral hemorrhage, there is an increase in the release of many cytokines, including IL-1, TNF- α and VEGF can activate the synthesis of S1P (32). After cerebral hemorrhage, the levels of WBC, fibrinogen, and CRP increase, which, in turn, may lead to an increase in systemic S1P release from platelets, WBC, and endothelial cells (the main source of cells) (33). S1P exerts various pathological and physiological functions by binding to the receptor family of its G protein coupled receptors, and different subtypes of S1PR play different roles in ICH (34). The S1P gradient between the tissue and systemic circulation, as well as the interaction between S1P-S1P1, are crucial for the expulsion of lymphocytes from the thymus and secondary lymphoid organs (SLO) (35). S1PR3 affects pro-inflammatory M1 polarization and activates the p38 MAPK pathway, which is involved in brain injury after ischemic stroke (36). Previous studies have found that S1P and S1PR are associated with impaired BBB function in various central nervous system (CNS) diseases (37). Elevated levels of S1P may also express a stronger inflammatory response to ICH, leading to a cascade of secondary injury reactions and impaired healing and recovery (30). It is uncertain whether the local production of S1P by immune cells directly leads to elevated serum levels in patients with ICH (38).

We also found a correlation between S1P level and ICH volume. A larger volume of ICH may trigger larger systemic inflammatory responses in the central nervous system and periphery, leading to an early increase in S1P levels after ICH. However, in the hierarchical analysis of location, we found a stronger correlation between S1P and bleeding volume in deep cerebral hemorrhage, which has stronger predictive value for disease severity. This seems to contradict other similar studies on intracerebral hemorrhage inflammation, and we consider that it may be due to the small sample size of lobar hemorrhage in this study, or the fact that patients with deep intracerebral hemorrhage have more severe diseases and excessive bleeding. This still requires further expansion of the sample size for in-depth exploration.

Given the important role of S1P in inflammatory response, S1PR has been widely studied as a potential therapeutic target (39). Fingolimod is a non-selective S1PR antagonist that attenuated neurological deficits by preventing lymphocyte egress from lymphoid organs in a rodent model of ICH and it entered clinical trials for the treatment of ICH (40). On the other hand, it can induce homing of lymphocytes during migration and even induce apoptosis of lymphocytes to exert immunosuppressive effects (33). Bobinger et al. found that administration of Siponimod as early as 30 min after ICH significantly reduced the number of spleen cells in the circulating blood of mice, leading to a downregulation in the number of spleen cells in the brain. This in turn alleviated the development of brain injury and neurological dysfunction after ICH (17). Fingolimod has also been shown to improve brain damage in ischemic stroke models by enhancing blood supply and neurological function, as well as protecting the BBB. It is currently undergoing phase II clinical trials for acute ischemic cerebrovascular disease (41, 42). The research data has shown that the volume of edema around the hematoma, phenylalanine levels, and circulating lymphocyte count in patients treated with Fingolimod are significantly smaller than those in the control group (43). However, there is no definitive research evidence regarding the optimal duration of treatment or the optimal administration time. Further studies are needed to ascertain whether S1P anti-therapies can improve outcomes after ICH.

Our research had some noteworthy limitations. First, being a retrospective study, it had the inherent limitations of such a design. Second, this was a single-center cohort study, and the sample size was not sufficiently large. In addition, another limitation of our study is the lack of biological curves for S1P. In Wang et al.’s study, it was found that plasma S1P levels significantly increased upon admission, peaked at day 5, declined at day 7, and were significantly higher than the control group within 7 days (44). On this basis, our study verified the increase of plasma S1P after ICH, and further explored the correlation between plasma S1P levels and some adverse clinical outcomes and other laboratory indicators. The location of ICH was used as a stratification to explore the impact of ICH location on the relationship between S1P and disease severity and prognosis. There is a certain degree of innovation. Therefore, we believe that our observations warrant further research on a larger scale to verify the ability of serum S1P levels to forecast disease severity and prognosis. We observed an increase in plasma S1P levels in the early stages of cerebral hemorrhage, which may play a crucial role in the pathophysiology of early ICH and could contribute to the emergence of new therapies. Understanding the mechanism of increased S1P generation in ICH and its multisystem signaling effects should be the focus of future work to strengthen our understanding of the prognosis and treatment of cerebral hemorrhage.

## Conclusion

In summary, we found that the S1P level at admission was associated with more severe disease and poor prognosis after ICH, suggesting that plasma S1P may become a new biomarker for ICH. Further research is needed to evaluate the role of S1P as a potential intervention target for cerebral hemorrhage.

## Data availability statement

The original contributions presented in the study are included in the article/Supplementary material, further inquiries can be directed to the corresponding author.

## Ethics statement

The studies involving humans were approved by the Ethics Committee of Zhengzhou University (Ethics Review No. 2023-KY-0242). The studies were conducted in accordance with the local legislation and institutional requirements. The participants provided their written informed consent to participate in this study.

## Author contributions

XY: Data curation, Formal analysis, Investigation, Methodology, Project administration, Writing – original draft, Writing – review & editing. KW: Investigation, Methodology, Project administration, Writing – original draft. PS: Data curation, Investigation, Project administration, Writing – review & editing. TZ: Data curation, Investigation, Project administration, Writing – review & editing. YX: Formal analysis, Investigation, Writing – review & editing. YC: Formal analysis, Methodology, Project administration, Validation, Writing – review & editing. YL: Methodology, Project administration, Supervision, Validation, Writing – review & editing. YY: Investigation, Supervision, Writing – review & editing. ZG: Investigation, Supervision, Writing – review & editing. RD: Conceptualization, Software, Writing – review & editing. LJ: Investigation, Supervision, Writing – review & editing. YJ: Funding acquisition, Methodology, Project administration, Resources, Supervision, Writing – review & editing.
